# Quality of life in irradiated patients with head and neck cancer: A preliminary study about the impact of prosthetic rehabilitation

**DOI:** 10.4317/jced.58209

**Published:** 2021-09-01

**Authors:** Frédéric Silvestri, Bérengère Saliba-Serre, Nicolas Graillon, Nicolas Fakhry, Michel Ruquet, Gérald Maille

**Affiliations:** 1 Faculté d’Odontologie, Aix-Marseille Université, 27 boulevard Jean Moulin, 13555 Marseille Cedex 5; Pôle Odontologie, UF des Soins Spécifiques, Hôpital Timone, Assistance Publique des Hôpitaux de Marseille, 264 rue Saint-Pierre, 13385 Marseille Cedex 5, France. EFS CNRS, Faculté des Sciences Médicales et Paramédicales, UMR 7268 ADES, Aix-Marseille Université, 51 boulevard Pierre Dramard, 13944 Marseille Cedex 15, FranceFaculté d’Odontologie, Aix-Marseille Université, 27 boulevard Jean Moulin, 13555 Marseille Cedex 5; Pôle Odontologie, UF des Soins Spécifiques, Hôpital Timone, Assistance Publique des Hôpitaux de Marseille, 264 rue Saint-Pierre, 13385 Marseille Cedex 5, France. EFS CNRS, Faculté des Sciences Médicales et Paramédicales, UMR 7268 ADES, Aix-Marseille Université, 51 boulevard Pierre Dramard, 13944 Marseille Cedex 15, France; 2 EFS CNRS, Faculté des Sciences Médicales et Paramédicales, UMR 7268 ADES, Aix-Marseille Université, 51 boulevard Pierre Dramard, 13944 Marseille Cedex 15, France; 3 Faculté des Sciences Médicales et Paramédicales, Aix-Marseille Université, 27 boulevard Jean Moulin, 13555 Marseille Cedex 5; Pôle PROMO, Service de Chirurgie Maxillofaciale Stomatologie et Chirurgie Orale, Hôpital de la Conception, Assistance Publique des Hôpitaux de Marseille, 147 boulevard Baille, 13005 Marseille, France; 4 Faculté des Sciences Médicales et Paramédicales, Aix-Marseille Université, 27 boulevard Jean Moulin, 13555 Marseille Cedex 5; Pôle PROMO, Service ORL et Chirurgie Cervico-faciale, Hôpital de la Conception, Assistance Publique des Hôpitaux de Marseille, 147 boulevard Baille, 13005 Marseille, France; 5 Faculté d’Odontologie, Aix-Marseille Université, 27 boulevard Jean Moulin, 13555 Marseille Cedex 5; Pôle Odontologie, UF des Soins Spécifiques, Hôpital Timone, Assistance Publique des Hôpitaux de Marseille, 264 rue Saint-Pierre, 13385 Marseille Cedex 5, France. EFS CNRS, Faculté des Sciences Médicales et Paramédicales, UMR 7268 ADES, Aix-Marseille Université, 51 boulevard Pierre Dramard, 13944 Marseille Cedex 15, France; 6 Faculté d’Odontologie, Aix-Marseille Université, 27 boulevard Jean Moulin, 13555 Marseille Cedex 5; Pôle Odontologie, UF des Soins Spécifiques, Hôpital Timone, Assistance Publique des Hôpitaux de Marseille, 264 rue Saint-Pierre, 13385 Marseille Cedex 5, France. EFS CNRS, Faculté des Sciences Médicales et Paramédicales, UMR 7268 ADES, Aix-Marseille Université, 51 boulevard Pierre Dramard, 13944 Marseille Cedex 15, France

## Abstract

**Background:**

Oral quality of life is of great importance in head and neck cancer, where each patient combines functional, social and esthetic needs. Our study aimed to evaluate the influence of prosthetic and/or maxillofacial rehabilitation on patients’ perceived oral quality of life.

**Material and Methods:**

The General Oral Health Assessment Index (GOHAI) was used in 28 patients with head and neck cancer who had undergone radiotherapy, recruited at La Timone University Hospital, Marseille, France, and who required prosthetic rehabilitation. The questionnaire was completed at three timepoints in the study: before insertion of the prosthesis (T0), then one week (T1) and three months after insertion (T2).

**Results:**

The percentage of patients with poor quality of oral health decreased from 96.4% to 64.3% between T0 and T1. Between T0 and T1, the mean score of the psychosocial component of the GOHAI increased from 14.28 ± 4.51 to 20.14 ± 5.20 and the mean functional component score increased from 9.32 ± 3.86 to 12.07 ± 4.04.

**Conclusions:**

Prosthetic rehabilitation appeared to have a positive impact on oral quality of life in our study subjects, particularly on social relations and self-esteem. Its influence on pain and discomfort remains to be clarified. This preliminary study gives a prospective view of the impact of prosthetic rehabilitation in patients with head and neck cancer who had undergone radiotherapy. Analysis of data yielded by cross-referencing of different questionnaires should make it possible to refine these results.

** Key words:**GOHAI, quality of life, head and neck cancer, prosthetic rehabilitation.

## Introduction

According to the technical report on the annual projection of mortality and incidence of cancer in France for 2017 (1), head and neck cancers represent 20100 new cases and a mortality of 6,900 individuals. Irradiation treatment, whether curative, palliative or adjuvant, is the main control tool in the therapeutic arsenal against head and neck cancers.

The World Health Organization defines quality of life as « individuals’ perception of their position in life in the context of the culture and value systems in which they live and in relation to their goals, expectations, standards and concerns. This concept is very largely influenced in a complex way by the physical health of the subject, psychological state, level of independence, social relationships as well as relation to the essential elements of his environment » (2). There are thus many different aspects to quality of life, one of which is oral quality of life. Oral health is an indicator of overall health, well-being and quality of life. It encompasses a range of diseases and conditions that include dental caries, periodontal disease, tooth loss, oral cancer, oral manifestations of HIV infection, oro-dental trauma, noma and birth defects such as cleft lip and palate (3).

In the context of cancer, multidisciplinary teams pay attention to the quality of the remaining lifetime of patients in their care. Head and neck cancers involve a functional crossroads and have a fundamental impact on patients’ perceived quality of life. Because of this, maxillofacial prosthetic dentistry has a place in the multidisciplinary approach (4,5).

In these situations, conventional fixed or removable prostheses are most frequently prescribed. In parallel, Sharaf *et al*. have shown that such prostheses are of great value in the early post-surgical stages and are an alternative when reconstruction is not feasible.(6,7) Several authors have described the improvement of quality of life through functional improvement . Few studies have observed the true impact of the components of oral quality of life (pain and discomfort, psychosocial aspect, functional aspect) and their change over time.

The objective of this prospective study was to highlight changes in oral quality of life reported by patients at specific timepoints after insertion of the prosthesis. We investigated whether prosthetic rehabilitation led to changes in oral quality of life, and to what extent its different components were affected.

## Material and Methods

-Recruitment

Patients were recruited from the odontology department of La Timone University Hospital, Marseille, France, between June 7, 2016 and May 28, 2019. They were referred for consultation for prosthetic rehabilitation by maxillofacial surgery department of Conception University Hospital and included in a clinical research protocol on changes in oral health after radiotherapy of the upper aerodigestive tract (ClinicalTrials.gov Identifier: NCT02866500). Each participant gave their written informed consent.

Our study population consisted of a sample of 28 individuals who met the following inclusion criteria.

• patients who had or previously had head and neck cancer, 

• patients who had undergone radiotherapy, 

• patients requiring fixed, removable or implant-supported prosthetic rehabilitation.

Patients who had not wear their prosthesis or who were unable to complete the questionnaires were excluded from the study.

-Study design

The General Oral Health Assessment Index (GOHAI) was used to evaluate oral health-related quality of life (OHrQol). This questionnaire, developed by Atchison and Dolan, has been widely used to evaluate oral health in clinical or epidemiological studies (8). It was initially validated in the United States and has since been validated in several languages, notably in French (9). Subjects were asked if they had always, often, sometimes, seldom or never experienced any of the cited problems in the past month. Questions were worded sometimes positively, sometimes negatively, so that respondents needed to reflect on their answers. Responses were scored on a scale ranging from 1 to 5.

The total score on the GOHAI ranges from 12 to 60. The higher the score, the better the quality of oral health (Table 1). The score obtained was classified into three categories.

**Table 1:**
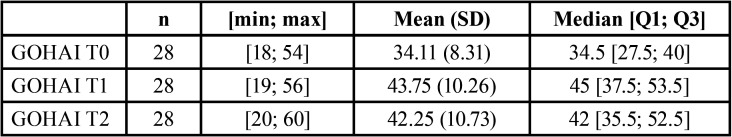
Numerical summary of the global GOHAI score at the different timepoints (n = 28).

- [12 to 50]: low score, poor oral quality of health 

- [51 to 56]: intermediate score, medium oral quality of health, 

- [57 to 60]: high score, good oral quality of health.

The 12 questions of the GOHAI cover three fields.

- Functional (eating, speaking, swallowing), corresponding to items 1 to 4, with a total score of 4 to 20, 

- Psychosocial (concerns, relational discomfort, appearance), corresponding to items 6, 7, 9, 10 and 11, with a total score of 5 to 25, 

- Pain and discomfort (drugs, sensitive gums, discomfort when chewing certain foods), corresponding to items 5, 8 and 12, with a total score of 3 to 15.

The patients’ age, sex, medical history and drug treatments were recorded. Oral examination was carried out for diagnosis and to determine the type of prosthetic rehabilitation required.

Patients were asked to answer the GOHAI questionnaire at three timepoints, T0, T1 and T2:

• T0: before insertion of the prosthesis,

• T1: one week after insertion,

• T2: 3 months after insertion.

During the study, the number of individuals decreased due to various factors, such as death, loss to follow-up, or because they did not receive a prosthesis. For this preliminary study, statistical analysis was carried out on the 28 patients followed from T0 to T2.

-Statistical analysis 

Detailed descriptive analysis was performed. Qualitative variables were presented as numbers and percentages (n, %). Quantitative variables were expressed as means and standard deviations (SD) and as medians sorted at the 25th and 75th percentiles (interquartile interval). Repeated measures analysis of variance was performed to assess differences in perceived oral health for each GOHAI score (global and the three fields) over time. When the sphericity assumption was violated, Greenhouse-Geisser or Huynh-Feldt corrections for departure from sphericity were applied. As some difference variables were not normally distributed, we used non-parametric tests, in particular post-hoc pairwise Wilcoxon signed rank tests, to examine differences between two timepoints. For all pairwise comparisons, the false discovery rate (FDR) approach was used to correct for multiple testing.

Statistical analyses were performed using SAS 9.4 software (SAS Institute Inc., Cary, NC, USA) and R Software version 3.5.2 (R Foundation for Statistical Computing, Vienna, Austria). All statistical tests were two-sided and the significance level used was 0.05.

## Results

-Descriptive statistics

•Characterization of the sample at T0

The population studied consisted of 28 patients aged 48.2 to 87.4 years of whom 11 (39,2%) were women and 17 were men (60.7%). The date of birth of one patient was not known, and for another the date of data collection at T0 was not given. The average age at the first consultation was 66.97 ± 11.64 years for men and 70.32 ± 10.87 years for women.

**Figure 1:**
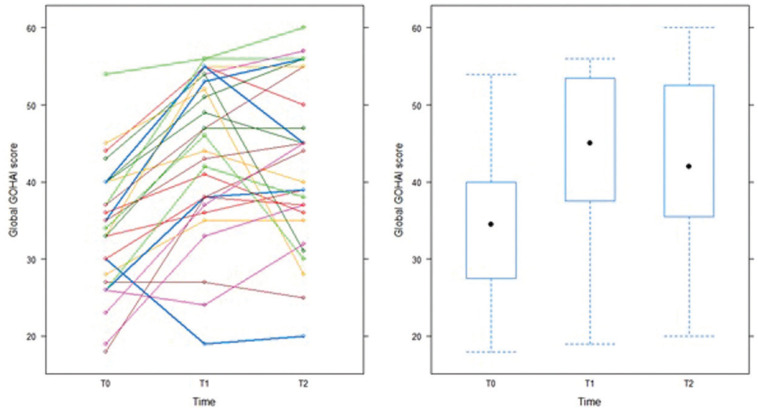
Individual profiles and boxplots showing change in the global GOHAI score from T0 to T2 (n = 28). The black circles indicate the median.

•Change in global GOHAI score and distribution by GOHAI class at the different timepoints (Fig. 1).

The global GOHAI score showed that 96.43% of patients reported poor oral quality of life before prosthetic rehabilitation (T0) compared with 64.29% one week after prosthesis insertion (T1). Three months after insertion (T2), 7.14% reported good oral quality of life. The mean global GOHAI score improved from 34.11 ± 8.31 to 43.75 ± 10.26 between T0 and T1. Between T1 and T2, the mean global score decreased to 42.25 ± 10.73 (Table 1).

•Change in the three GOHAI subscores 

The mean score for the pain and discomfort component of the GOHAI increased between T0 and T1 from 10.50 ± 2.69 to 11.53 ± 3.01. Between T1 and T2, it decreased to 11.46 ± 2.43.

The mean score for the functional component increased between T0 and T1 from 9.32 ± 3.86 to 12.07 ± 4.04. Between T1 and T2, it decreased to 11.82 ± 4.17.

**Figure 2:**
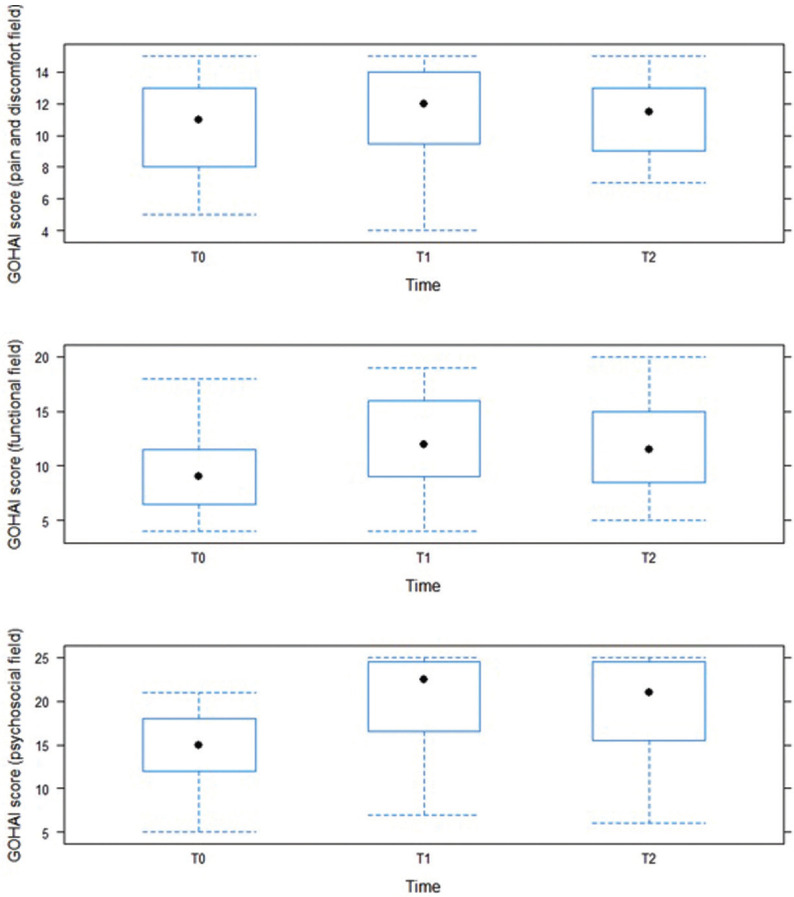
Boxplot representations of the three GOHAI subscores at the different timepoints (n = 28).

The mean score for the psychosocial component increased between T0 and T1 from 14.28 ± 4.51 to 20.14 ± 5.20. Between T1 and T2, it decreased to 18.96 ± 6.04 (Table 2, Fig. 2).

**Table 2:**
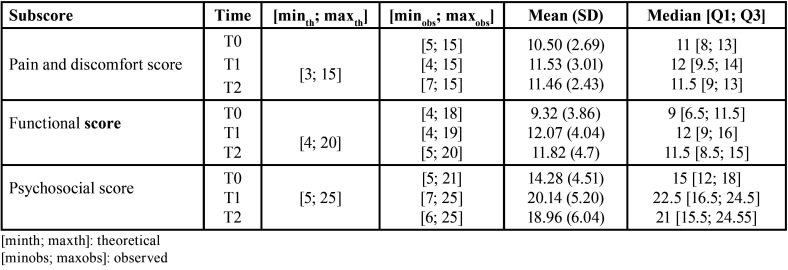
Numerical summary of the GOHAI subscores for the different timepoints (n = 28).

Whiskers extend to the extreme values. [min; max] theoretical values were [3; 15] for the pain and discomfort field, [4; 20] for the functional field and [5; 25] for the psychosocial field.

-Analytical statistics

The one-way repeated-measures analysis of variance was performed to test whether the mean GOHAI score and the subscores differed according to timepoint. There was a statistically significant effect of time at the 0.05 level for each score (Table 3). Results of post-hoc pairwise tests to compare each score between two timepoints are given in Table 4.

**Table 3:**
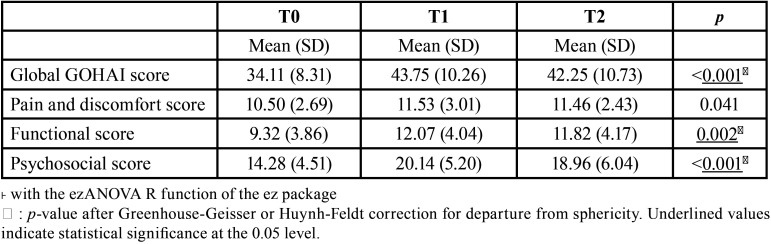
Summary statistics of the global GOHAI score and subscores at different timepoints and results of one-way repeated measures ANOVA˫ (n = 28).

**Table 4:**
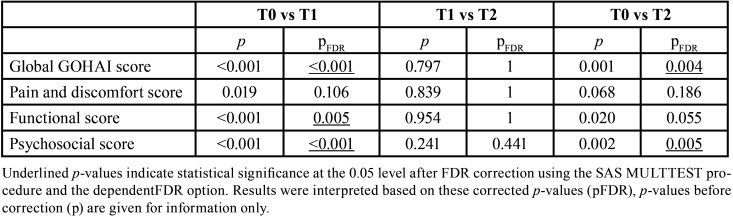
Results of post-hoc pairwise tests comparing each GOHAI score between two timepoints.

For the global GOHAI score, a significant difference was observed between T0 and T1 (pFDR <0.001) and between T0 and T2 (pFDR = 0.004). No difference was found between T1 and T2.

For pain and discomfort, no significant difference was observed, whether between T0 and T1, T1 and T2, or T0 and T2.

A significant difference in the functional subscore was observed between T0 and T1 only (pFDR = 0.005).

For the psychosocial subscore, significant differences were observed between T0 and T1 (pFDR <0.001) and between T0 and T2 (pFDR = 0.005).

## Discussion

-Sample

Data analysis focused on the sample of 28 individuals followed until the T2 timepoint. The unequal sex distribution of the patients corresponds to the incidence of head and neck cancers. Patients included in this preliminary study have undergone radiotherapy (60 Gy, focused on the oral cavity) and chemotherapy. However, the sample size did not allow us to carry out statistical analysis of the results according to primary tumor location or size of surgical excision. As each of these factors affects oral quality of life, sample size should be increased (10-13). Some authors have shown that patients may require up to 6 months to adjust to their prosthesis, and this may lead to bias in the discussion, particularly for the functional component (12). For this reason, patients who report to not wear their prosthesis were excluded from the sample. All patients included in this study were rehabilitated with a removable prosthesis but the sample size did not allow to separate partial removable prosthesis from complete prothesis.

The study should be continued for one year after oral rehabilitation. Patients who weared prothesis before treatment and patients who never weared one could be then studied separately.

-Change in the global GOHAI score 

Study of change in the global GOHAI score showed improvement in perceived oral quality of life, in particular between T0 and T1. This can be interpreted as an immediate impact of prosthetic rehabilitation: 96.43% poor quality of life at T0 compared with 64.29% at T1. Figure 1 shows an increase in the score between T0 and T1 and therefore an improvement in oral quality of life. This improvement appears to be maintained, since at T2 7.14% of patients reported good oral quality of life and 17.86% medium quality of life.

We can therefore provide a preliminary response, indicating that prosthetic rehabilitation had a positive impact on oral quality of life.

The functional component score (Table 3) increased between T0 and T1 from 9.32 ± 3.86 to 12.07 ± 4.04), then stabilized between T1 and T2 (11.82 ± 4.17). There was a significant difference between the scores from T0 to T1 but not between T1 and T2.

Increased ability to chew through prosthetic rehabilitation explains the immediate functional improvement.

However, the adverse effects of ionizing radiation, such as radio-induced xerostomia, present in at least 50% of individuals suffering from head and neck cancers, have consequences on different functions (swallowing, speech, chewing, etc.). They also cause burning sensations and pain (14). These side effects all make the use of maxillofacial prostheses more difficult. This may also explain the lack of improvement in the score of the functional component of oral quality of life over time.

John *et al*. showed that adaptation to prosthetic rehabilitation is only achieved between 6 and 12 months after insertion of the prosthesis (15). Insufficient follow-up duration of prosthesis wearing in our patients could be one of the reasons for the absence of significant change in the scores. In addition, Schweyen *et al*. observed in their study that individuals with removable partial or full dentures admitted that they wore their dentures only occasionally (12). This prolongs the time necessary for the patient to adapt to their prosthesis.

The pain and discomfort component in the sample increased from 10.50 ± 2.69 to 11.53 ± 3.01 between T0 and T1, then to 11.46 ± 2.43 at T2 (Table 4). The scores for this component showed no significant differences between the different timepoints. Being fitted with a dental or maxillofacial prosthesis does not modify the pain problems, and this may account for a relatively sTable score several weeks after prosthesis insertion. Terkawi *et al*. observed that 42% of a group of individuals who had undergone excision surgery for head or neck cancer reported chronic pain up to 6 years after completion of their treatment (16).

Some studies have shown that the quality of life as perceived by individuals, whether ill or in good health, can be influenced by the psychosocial aspect or by their psychological disposition (17). However, few studies have specifically addressed change in this component over time.

 In our study, scores for the psychosocial component increased from T0 (14.28 ± 4.51) to T1 (20.14 ± 5.20), then stagnated at T2 (18.96 ± 6.04) (Table 3). The absence of prosthetic rehabilitation at T0, followed by prosthesis insertion at T1, obviously have a significant impact on the score for this component. It showed that removable prosthesis could increased immediately oral-related quality of life : this marked increase in the score of psychosocial component (40%) could constitute a path to deeply understand quality of life improvement mechanisms in the few days after surgery. In addition, the contribution of prosthetic rehabilitation remains stable since it is mainly linked to subjective parameters in the daily lives of individuals. This is supported by a study by Moroi *et al*. which showed that 24% of men and 31% of women reported that their physical appearance had a negative influence on their quality of life (18). For this component, these data may indicate that prosthetic rehabilitation fulfills its purpose from T1 onwards. Therefore, the scores do not show any significant positive or negative development over time. The corrections made on pairwise comparisons calculated following multiple comparisons concord with this: there was a significant difference in the results between T0 and T2, but not between T1 and T2 (Table 4).

This preliminary study provides a prospective view of the impact of prosthetic rehabilitation in patients with head and neck cancer who had undergone radiotherapy. The originality of this article results in methodology : same practioner used same protocols to manage prosthetic rehabilitations, patients follow-up and data collections. Methodological biases could be reduced by removing parameters related to practitioners. It demonstrated an increase in the GOHAI score, and more particularly in the score of the psychosocial component, between the different timepoints. Moreover, it showed that removable rehabilitations could increase oral-related quality of life and play a major role in immediate rehabilitations of maxillofacial substance losses. It also highlights the need to plan prosthetic rehabilitation before surgical treatment in order to solve problems of nutrition and facilitate the acceptance of oncologic treatment. In order to refine our results, this study will be supplemented by cross-referencing of data collected from several specific questionnaires used in oncology (GOHAI, European Organization for Research and Treatment of Cancer QLQ-C30 and the H&N35 module) (19,20).

## Conclusions

Development of this protocol should enable us to better quantify the oral quality of life of individuals by analyzing elements related to the context of treatment of head and neck cancers by radiotherapy, and to anticipate the functional, esthetic and social issues that patients will encounter. By taking into account factors of influence such as the type of cancer and its location, and the type of excision surgery, it seems possible to plan for therapeutic management in a personalized manner (surgery, prosthetic rehabilitation). In connection with the multidisciplinary care team, treatment protocols could be accurately established to be more efficient, allowing an increase in quality of life of patients after prosthetic rehabilitation.
